# Detection of Stable Elite Haplotypes and Potential Candidate Genes of Boll Weight Across Multiple Environments via GWAS in Upland Cotton

**DOI:** 10.3389/fpls.2022.929168

**Published:** 2022-06-13

**Authors:** Zhen Feng, Libei Li, Minqiang Tang, Qibao Liu, Zihan Ji, Dongli Sun, Guodong Liu, Shuqi Zhao, Chenjue Huang, Yanan Zhang, Guizhi Zhang, Shuxun Yu

**Affiliations:** ^1^College of Advanced Agriculture Sciences, Zhejiang A&F University, Hangzhou, China; ^2^The Key Laboratory for Quality Improvement of Agricultural Products of Zhejiang Province, Zhejiang A&F University, Hangzhou, China; ^3^Key Laboratory of Genetics and Germplasm Innovation of Tropical Special Forest Trees and Ornamental Plants (Ministry of Education), College of Forestry, Hainan University, Haikou, China; ^4^Institute of Industrial Crops, Shandong Academy of Agricultural Sciences, Jinan, China; ^5^Huanggang Academy of Agricultural Sciences, Huanggang, China

**Keywords:** SNP, boll weight, association mapping, candidate genes, MAS

## Abstract

Boll weight (BW) is a key determinant of yield component traits in cotton, and understanding the genetic mechanism of BW could contribute to the progress of cotton fiber yield. Although many yield-related quantitative trait loci (QTLs) responsible for BW have been determined, knowledge of the genes controlling cotton yield remains limited. Here, association mapping based on 25,169 single-nucleotide polymorphisms (SNPs) and 2,315 insertions/deletions (InDels) was conducted to identify high-quality QTLs responsible for BW in a global collection of 290 diverse accessions, and BW was measured in nine different environments. A total of 19 significant markers were detected, and 225 candidate genes within a 400 kb region (± 200 kb surrounding each locus) were predicted. Of them, two major QTLs with highly phenotypic variation explanation on chromosomes A08 and D13 were identified among multiple environments. Furthermore, we found that two novel candidate genes (*Ghir_A08G009110* and *Ghir_D13G023010*) were associated with BW and that *Ghir_D13G023010* was involved in artificial selection during cotton breeding by population genetic analysis. The transcription level analyses showed that these two genes were significantly differentially expressed between high-BW accession and low-BW accession during the ovule development stage. Thus, these results reveal valuable information for clarifying the genetic basics of the control of BW, which are useful for increasing yield by molecular marker-assisted selection (MAS) breeding in cotton.

## Introduction

Cotton has an ancient history of cultivation dating back seven thousand years or more according to the oldest archeological evidence, which was found in Pakistan (Rajpal et al., 2016). Subsequently, the invention of the cotton gin in the late 18^th^ century caused massive growth in cotton production, and cotton gradually became an important cash crop (Sunilkumar et al., 2006). Previous studies have suggested that allotetraploids emerged approximately 1.5 million years ago (MYA) through a single allopolyploidization event in a propagule resembling diploid cotton (*Gossypium herbaceum* L.) that dispersed across the Atlantic Ocean from Africa to the New World and subsequently hybridized with a resembling diploid cotton (*Gossypium raimondii*) and produced upland cotton after long-term evolution (Wendel, 1989; Sunilkumar et al., 2006; Liu et al., 2015). Currently, upland cotton has become a predominant cotton species in global cotton commerce, with ~ 27 million metric tons produced per year. In addition, it also provides natural fiber for the textile industry, which has high yield and wider adaptation (Chen et al., 2007). In recent years, due to population growth, climate change, and the challenges associated with maintaining the grain-cotton balance in farmlands, the cotton planting area has decreased. Therefore, the urgent need to increase cotton production is particularly important.

The application of quantitative trait locus linkages or QTL-related molecular markers of target traits by MAS can prevent environmental interference and improve breeding efficiency (Yin et al., 2003). The study of QTLs in cotton has focused mainly on yield and fiber quality component traits (Said et al., 2015). Cotton yield component traits include fruit branch number (FBN), lint percentage (LP), boll number per plant (BN), boll weight (BW), and seed index (SI), which were controlled by QTLs and environmental factors. Among these traits, BW is more stably inherited and has relatively high heritability (Fan et al., 2018; Liu et al., 2018; Zhang et al., 2019b; Gu et al., 2020; Zhu et al., 2021). In the past three decades, BW has been widely used for quantitative genetics studies, and a great number of studies have been conducted to identify genetic locus for BW distributed on almost all chromosomes via classic linkage maps and genome-wide association studies (GWAS) using cotton panels; over 170 QTLs for BW have been discovered (Said et al., 2015; Liu et al., 2018; Wang et al., 2019b; Zhu et al., 2021). By using F_2_ and F_2:3_ populations derived from an upland cotton intraspecific cross (Simian3 x TM-1), several yield-related QTLs were identified by simple sequence repeat (SSR) and random amplified polymorphic DNA (RAPD) markers, and common QTLs explaining 15.6% of the phenotypic variation (PV) were identified for BW and 100-seed weight on chromosome A09 (Yin et al., 2002). Wang et al. (2015) constructed a linkage map, which included 178 loci spanning 2016.44 cM, and a total of 19 QTLs for BW were detected on seven chromosomes; two QTLs were identified in more than two environments. In addition, a previous study involving 356 cotton accessions identified four favorable alleles for BW by a GWAS panel (Mei et al., 2013). The elucidation of the genetic architecture of BW can provide strong theoretical support for breeders to increase cotton production. However, there still exists inadequacy in previous research, such as the use of low-density linkage maps constructed based on traditional molecular markers, incomplete genetic information of the reference genome, and rough resolution of the mapping interval, resulting in candidate genes that could not be directly identified. SNP markers could be more effectively to explore the genetic structure in important agronomic traits in biparental map-based cloning and association analysis based on their highly polymorphism, wide distribution, and low research costs (Van Tassell et al., 2008; Ganal et al., 2009). Along with the reduction in high-throughput sequencing costs, a great quantity of SNP markers has been extensive development (Michael et al., 2018; Sun et al., 2020), leading to more candidate genes can be identified by QTL mapping and GWAS through SNP markers (Zhou et al., 2020; Li et al., 2021). In recent years, candidate genes for yield component traits in cotton have been wide-ranging explored in genetic studies with SNP markers rather than traditional molecular markers. For example, Zhang et al. (2016) constructed a high-density genetic map containing 5,521 SNP markers developed with a recombinant inbred line (RIL) population in 11 environments, and 344 candidate genes for BW were annotated. In addition, Fang et al. (2017) employed whole-genome resequencing using 1,871,401 high-quality SNP markers in 258 diverse accessions and discovered that the candidate gene *Gh_D08G0312* may be a key gene determining cotton yield. Moreover, two candidate genes associated with lint percentage were uncovered using 276 upland cotton accessions with 10,660 SNPs in multiple environments; these genes were highly expressed during ovule and fiber development, indicating that they may play important roles in influencing LP (Song et al., 2019). Although QTLs for yield component traits have been extensively explored in upland cotton, compared to those in important crops such as rice and maize, few candidate genes have been identified.

For this study, to gain better insight into the genetic basics of BW, specific locus amplified fragment sequencing (SLAF-seq) was taken as for whole-genome identification of SNPs and InDels in a natural population. PV for BW in nine environments was evaluated across four representative agroecological regions. In addition, several QTLs and candidate genes were further identified by a GWAS. This study provides information regarding a valuable cotton germplasm potentially useful for MAS in cotton breeding practice for raising yield in upland cotton.

## Materials and Methods

### GWAS Population and Field Experiments

A total of 290 elite upland cotton accessions were obtained from CRICAAS (http://www.cricaas.com.cn/). Among these accessions, 263 (90.7%) representative cultivars were collected from four major cotton production regions of China: Northern-Specific Early-Maturity region (NSER), Yellow River region (YRR), Yangtze River region (YZRR), and Northwest Inland region (NIR). The remaining 27 (9.3%) cultivars were introduced from six different countries (USA, Azerbaijan, Israel, Kyrgyzstan, Tajikistan, and Uzbekistan). Complete GWAS population material of each accession is shown in Supplementary Table S1. A natural population of 290 upland cotton accessions was planted at Anyang (36 08'N, 114 48'E) in three consecutive years (2014, 2015, and 2016) (E1: Anyang-2014, E2: Anyang-2015, and E3: Anyang-2016); Shihezi (44 31'N, 86 01'E) in three consecutive years (2014, 2015, and 2016) (E4: Shihezi-2014, E5: Shihezi-2015, and E6: Shihezi-2016); Huanggang (30 57'N, 114 92'E) in 2 years (2016 and 2021) (E7: Huanggang-2016 and E8: Huanggang-2021); and Sanya (18 36'N, 109 17'E) from 2020 to 2021 (E9: Sanya-2020-2021). Each environment was conducted with a randomized complete block for three replications.

### Phenotyping and Statistical Analysis of BW

In total, 20 mature cotton bolls were randomly harvested from the middle branches and dried under sunlight for 2 days in each line. The phenotypic data from all the environments were analyzed with the base packages of R software (version: 3.5.0), and the correlation analysis results were exhibited with the “corrplot” (Wei et al., 2017). The broad-sense heritability (*H*^2^) of BW progressed with the “sommer” (Covarrubias-Pazaran, 2016). In addition, the BLUP value of boll weight in the nine environments for the GWAS analyses was conducted by the “lme4” (Bates et al., 2014).

### Genome Sequencing and Variation Detection

We collected young leaves at seedling stage of each line for genotyping. The SLAF-seq libraries were constructed for each accession based on the restriction enzymes *Rsa* I and *Hae* III (New England Biolabs, NEB). All accessions were genotyped with the Illumina HiSeq2500 platform. The detailed protocols used for library preparation and sequencing using the SLAF strategy have been described previously (Li et al., 2017). The quality control process was employed by Trimmomatic (version: 0.32) (Bolger et al., 2014), and then, the filter reads were aligned to reference genomes of the three upland cotton accessions (“TM-1,” “CRI24,” and “NDM8”) by using BWA (version: 0.7.17) (Li and Durbin, 2009; Yu et al., 2021). The high-quality SNPs and InDels were detected using Genome Analysis Toolkit software (version: 3.8) (McKenna et al., 2010).

### GWAS and Genetic Diversity Analysis

For GWAS analysis, we first filtered the SNPs and InDels with a minor allele frequency (MAF) less than 0.05 and a missing rate greater than 80%. Second, population structure was calculated as the covariate to reduce false positives (Supplementary Figure S1). Finally, the linear mixed mode in GEMMA (version: 0.98.3) (Zhou and Stephens, 2012) was used for discovering the significant locus by high-quality markers and BW values from each individual environment. The -log_10_(*P*) value was 4.43, which was used as 1/n (n = total number of SNPs and InDels in the GWAS panel) according to the Bonferroni-corrected method. The phenotypic variation explained (PVE) of each marker was calculated by the formula as follows: PVE = [2β^2^ × MAF × (1 – MAF)] / [2β^2^ × MAF × (1 – MAF) + ((se(β))^2^ ×2 × N × MAF × (1–MAF))], where β and MAF were obtained by the GEMMA software, and N represented the sample size according to previous reports (Shim et al., 2015). The R package “qqman” was used to generate Manhattan plots (Turner, 2014). The 290 accessions were split into three populations based on the release years, including cultivars released before the 1980s, cultivars bred within the 1980s−2000s, and cultivars bred after the 2000s; VCFtools (version: 0.1.16) was used to estimate nucleotide diversity (π) (Danecek et al., 2011) in the three populations. LD block analysis was conducted with the “LDheatmap” (Shin et al., 2006) to find existing LD blocks.

### Haplotype Analysis and Candidate Gene Identification

Haplotype analysis of associated markers on chromosomes A08 and D13 was conducted based on the phenotypic values and genotype data, and box plots were created using the R package “ggplot2” (Wickham, 2011). Candidate BW-related genes were identified and annotated on the basis of the “TM-1” genome released from COTTONGENE (https://www.cottongen.org/), which was in the upstream and downstream of 200 kb regions by significant markers according to previous reports (Su et al., 2018; Wang et al., 2019a). GO enrichment was performed on the agriGO to identify the enriched pathways by using default parameters (Tian et al., 2017).

### Gene Expression Level Analysis

The expression patterns in *G. hirsutum* L. “TM-1” and “CRI12” at the ovule development stage (10 days post-anthesis (DPA), 20 DPA, 30 DPA, and 40 DPA) were analyzed using the published RNA-seq dataset PRJNA248163 (Fang et al., 2017). The TPM values were determined using GFOLD software (version: 1.1.4) (Feng et al., 2012). We further performed qRT-PCR analysis. All gene-specific primers used in this study were designed using Primer3 (version: 0.4.0); they are listed in Supplementary Table S2. Seeds of upland cotton (G. *hirsutum* cv. “TM-1” and “CRI16”) were planted at Zhejiang A&F University in Hangzhou. Flowers were tagged on the day of anthesis. We collected bolls at 0, 5, 15, 20, and 25 DPA, and then, the young seeds with fibers were stripped of hulls, frozen in liquid nitrogen, and stored at −80^circ^C. Total RNA was extracted from the frozen 0, 5, 15, 20, and 25 DPA fibers and ovule using the MolPure® Plant Plus RNA Kit (Yeasen, Shanghai, China), and cDNA was synthesized using the MonScript™ RTIII Super Mix with dsDNase (Monad, Shanghai, China). Then, real-time PCR was performed to identify transcript levels using LightCycler 480 II PCR System (Mannheim, Germany) and MonAmp™ ChemoHS qPCR Mix (Monad, Shanghai, China). The 2^−Δ*ΔCT*^ method was applied to analyze the gene transcript abundance with three biological replicates (Livak and Schmittgen, 2001). Data visualization for qRT-PCR and RNA-seq was performed using custom R scripts.

## Results

### Detection of SNPs and InDels in Cotton Genome

**Figure 1:**
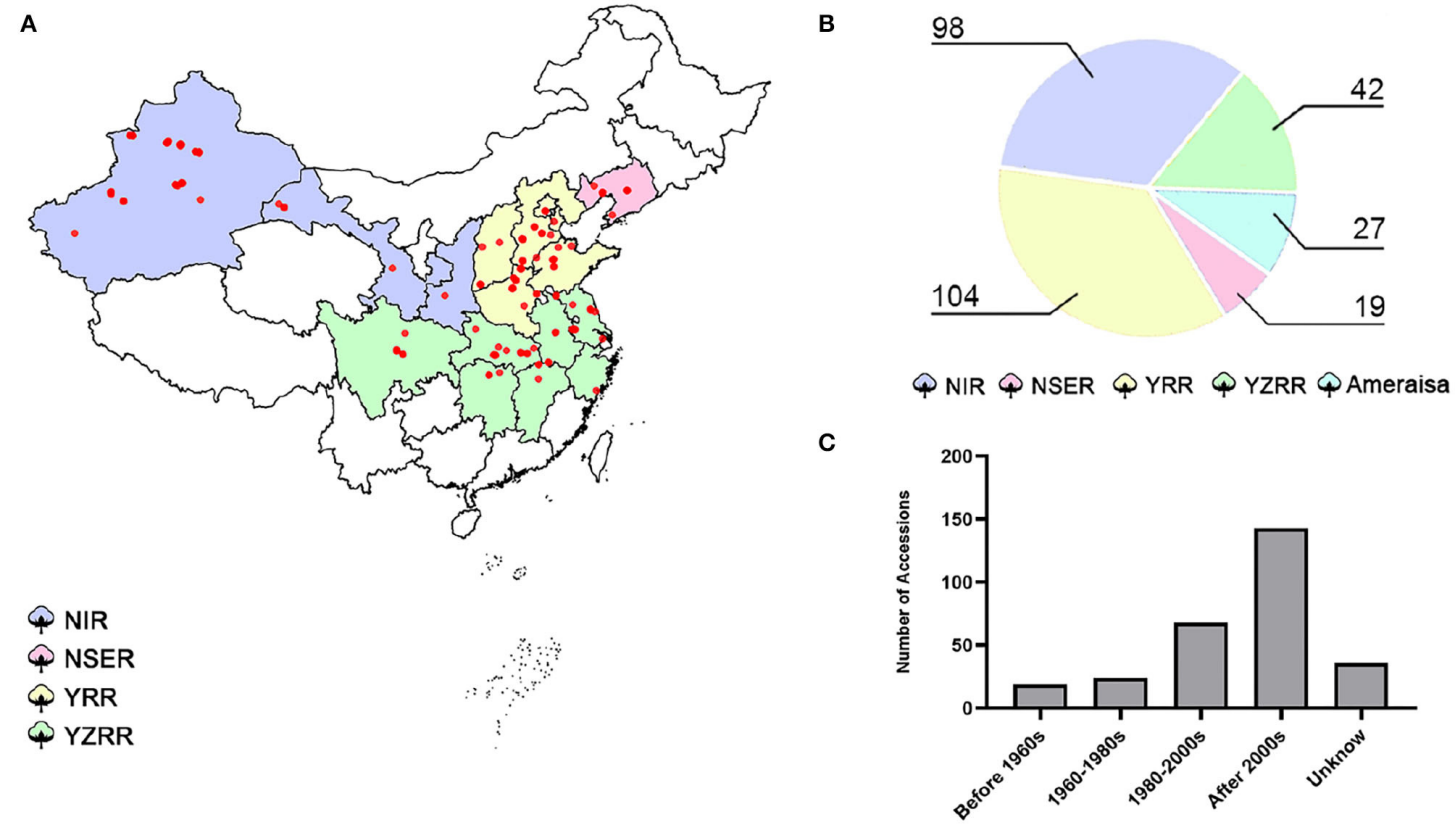
Map of the 290 cotton accessions. **(A)** Geographic distribution of the natural population; each accession is represented by a dot. **(B)** Pie chart of the proportions of diverse cotton-growing areas in 290 accessions. NIR: Northwest Inland region in China; NSER: Northern-Specific Early-Maturity region; YRR: Yellow River region; YZRR: Yangtze River region; and Amerasian: 27 accessions primarily introduced from six different countries (USA, Azerbaijan, Israel, Kyrgyzstan, Tajikistan, and Uzbekistan). **(C)** Breeding stage distribution of the GWAS panel; Unknown: accessions that were not found among the pedigrees.

A total of 290 cotton accessions (Supplementary Table S1) were selected from a wide global distribution, spanning over 100 years of cotton breeding, and genotyped using the SLAF-seq approach (Figure 1). To identify high-quality SNPs and InDels, we compared the mapping rates across seven high-quality published reference genomes from multiple research communities (Yu et al., 2014; Hu et al., 2019; Wang et al., 2019a; Yang et al., 2019; Chen et al., 2020; Huang et al., 2020; Ma et al., 2021). The number of SLAF reads with mapping rates ranging from 98.62 to 98.93% revealed no evidence of a significant difference, while HAU_v1 showed the largest number of high-quality SNPs and InDels (Supplementary Table S3). Thus, we selected HAU_v1 as a reference for further GWAS. A final set of 25,169 SNPs and 2,315 InDels were obtained with a MAF greater than 0.05 and missing data less than 20% in GWAS population. The mean marker density was one per 80.3 kb in the At subgenomes and one per 81.8 kb in the Dt subgenomes. Moreover, chromosome A06 possesses the highest number of markers (3,003 SNPs and 178 InDels), followed by chromosome A08 (2,827 SNPs and 189 InDels), and the smallest number of markers was observed on chromosome D03 (403 SNPs and 58 InDels) (Supplementary Figure S2).

### PV of BW

**Figure 2:**
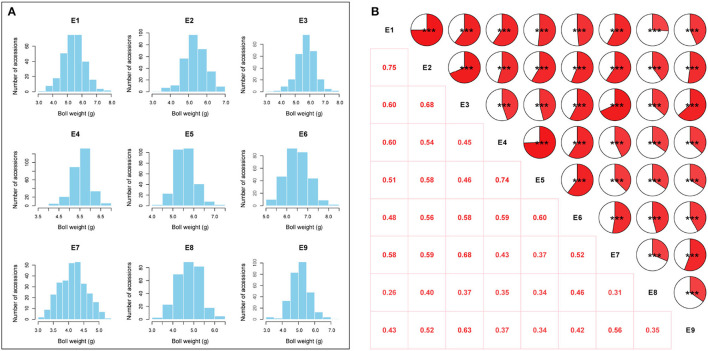
Phenotypic variation analysis of boll weight. **(A)** Distributions of the mean values for boll weight in nine environments (E1: Anyang-2014, E2: Anyang-2015, E3: Anyang-2016, E4: Shihezi-2014, E5: Shihezi-2015, E6: Shihezi-2016, E7: Huanggang-2016, E8: Huanggang-2021, and E9: Sanya-2020-2021). **(B)** Correlation analysis of boll weight in nine environments (***P < 0.001, **P < 0.01, and *P < 0.05).

The BW of 290 upland cotton accessions in nine environments followed an approximately normal distribution according to Shapiro–Wilk tests (Table 1). The frequency distributions of BW in the natural population are summarized in Figure 2A. The lowest average BW was 3.08 g in E7, and the highest average BW was 8.21 g in E6, with an average variation from 4.16 ± 0.44 to 6.48 ± 0.57 across the nine environments, suggesting extensive PV in the association panel (Table 1). The correlation analysis for BW exhibited relatively high positive correlations between environments (*P* < 0.001), with Pearson's correlation coefficients ranging from 0.26 to 0.75 (Figure 2B). On the contrary, a two-way ANOVA showed that genotypic variance (G) and the genotype-by-environment variance (G × E) had significant effects on BW (*P* < 0.001). This finding confirmed that a large number of genetic variations existed in the natural population. The *H*^2^ for BW was calculated as 69.65%, indicating that BW was mainly affected by the genotype, which was suitable for making further efforts association analysis (Supplementary Table S4).

**Table 1:** Phenotypic variation of BW in the natural populations.

**Environment**	**Minimum**	**Maximum**	**Mean**	**SD**	**Shapiro–Wilk *P* value**
E1 (Anyang-2014)	3.43	7.61	5.44	0.73	0.86
E2 (Anyang-2015)	3.30	6.93	5.35	0.60	0.65
E3 (Anyang-2016)	3.42	7.81	5.72	0.63	0.05
E4 (Shihezi-2014)	3.87	6.94	5.55	0.45	0.00
E5 (Shihezi-2015)	4.03	7.15	5.57	0.47	0.09
E6 (Shihezi-2016)	3.97	8.21	6.48	0.57	0.01
E7 (Huanggang-2016)	3.08	5.38	4.16	0.44	0.40
E8 (Huanggang-2021)	3.22	6.48	4.76	0.57	0.63
E8 (Sanya-2020-2021)	3.24	7.07	5.11	0.58	0.13

### GWAS of BW in Upland Cotton

**Figure 3:**
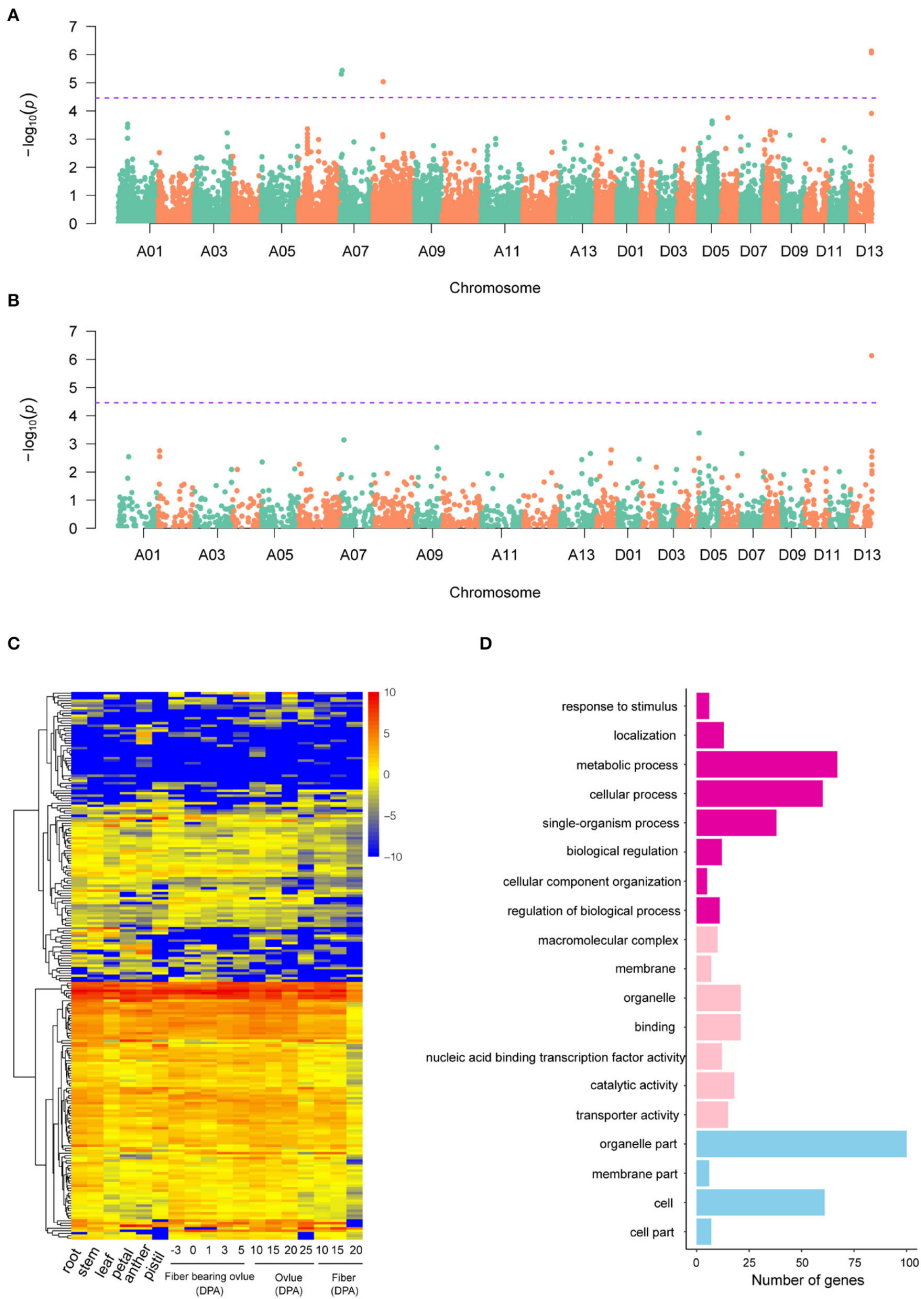
GWAS results of SNP and InDel markers and candidate gene analysis. **(A,B)** Manhattan plots of BW-BLUP for SNPs and InDels, respectively; significant BW-associated markers are distinguished by purple lines. **(C)** Heatmap of candidate gene expression patterns in 18 cotton tissues. **(D)** GO analysis of candidate genes associated with boll weight. The chart of purple, pink, and blue represented biological process, molecular function, and cellular component, respectively.

A GWAS of boll weight was performed with a linear mixed model (LMM) (Figures 3A,B and Supplementary Figures S3, S4). In total, 19 significant elite alleles with 16 SNPs and three InDels were identified on six chromosomes (A06, A07, A08, D01, D07, and D13) across nine individual environments and BW-BLUP values. Each allele explained 5.58 to 10.95% of the PV, and the -log_10_(*P*) values ranged from 4.53 to 6.13 (Table 2). A total of six loci were identified in at least two environments, and two major QTLs flanked by four alleles (rsA08_30171616, rsD13_60955253, rsD13_60955261, and rsD13_60955462) were further associated with BW-BLUP values (Table 2). Among them, one QTL significantly associated with a SNP (-log_10_(*P*) = 5.04) on chromosome A08 explained 9.38% of the PV. Notably, another major QTL region on chromosome D13 (60,820,223–60,955,462) was stably detected in six environments, and the BW-BLUP values were based on two SNPs and an InDel. The PV explained and -log_10_(*P*) values ranged from 10.32 to 10.95% and 6.06 to 6.13, respectively.

**Table 2:** List of significant markers (SNPs and InDels) associated with boll weight.

**Marker**	**Marker type**	**Chromosome**	**Position**	**Major allele**	**Minor allele**	***P* value**	***R^**2**^***	**Environment**
rsGhir_A06_26390257	SNP	A06	26,390,257	T	G	6.84E-06	6.67	E2
rsGhir_A06_26390265	SNP	A06	26,390,265	G	A	2.07E-05	5.92	E2
rsGhir_A06_26390284	SNP	A06	26,390,284	G	A	2.43E-05	6.04	E2
rsGhir_A06_26390468	SNP	A06	26,390,468	A	C	2.97E-05	5.58	E2
rsGhir_A06_26390491	SNP	A06	26,390,491	A	G	2.23E-05	6.09	E2
rsGhir_A06_32168831	Indel	A06	32,168,831	G	GT	2.14E-05	6.81	E8
rsGhir_A07_4798628	SNP	A07	4,798,628	G	A	4.91E-06	8.36	E7, BLUP
rsGhir_A07_6937342	SNP	A07	6,937,342	C	T	3.66E-06	8.57	BLUP
rsGhir_A07_6937395	SNP	A07	6,937,395	C	T	3.66E-06	8.38	BLUP
rsGhir_A07_9574709	SNP	A07	9,574,709	C	G	2.94E-05	7.69	E4, E5
rsGhir_A08_30171616	SNP	A08	30,171,616	A	G	9.20E-06	9.38	E6, E9, BLUP
rsGhir_D01_1229290	SNP	D01	1,229,290	A	G	8.35E-07	9.25	E8
rsGhir_D01_1229442	SNP	D01	1,229,442	T	C	1.94E-06	9.01	E8
rsGhir_D07_19492198	SNP	D07	19,492,198	G	A	2.95E-05	6.71	E1
rsGhir_D13_59526001	SNP	D13	59,526,001	G	C	2.41E-05	6.19	E4, E5
rsGhir_D13_60955253	Indel	D13	60,955,253	A	AT	7.40E-07	10.95	E1, E2, E3, E4, E5, E6, BLUP
rsGhir_D13_60955261	SNP	D13	60,955,261	G	T	7.51E-07	10.88	E1, E2, E3, E4, E5, E6, BLUP
rsGhir_D13_60955462	SNP	D13	60,955,462	A	G	8.73E-07	10.32	E1, E2, E3, E4, E5, E6, BLUP
rsGhir_D13_62059670	Indel	D13	62,059,670	GC	G	4.99E-06	6.06	E3

### Analysis of Candidate Genes Associated With BW

Potential candidate genes linked to 19 significant BW-associated markers were extracted based on the “TM-1” reference genome (Wang et al., 2019a). A total of 225 candidate genes were identified for BW, with most genes distributed on chromosome D13 and only one candidate gene located on chromosome A08 within the 400 kb genome region (Supplementary Table S5). Then, we identified orthologs for 225 candidate genes based on sequence similarity analysis by comparing the candidate genes to the *Arabidopsis thaliana* reference genome, which included 215 annotated genes and 10 novel genes (Supplementary Table S5). Furthermore, the expression levels of the 225 genes exhibited extensive variation among different cotton tissues representing vegetative growth processes, ovule developmental stages, and the primary fiber developmental stages of initiation, elongation, and secondary wall biosynthesis. The expression patterns of candidate genes were categorized into three groups, referred to here as lineages I, II, and III, based on similarities among the expression profiles (Figure 3C). Gene Ontology (GO) analysis found that a large proportion of genes (33.22%) had unknown functions, but most of the candidate genes were involved in metabolic processes (42.68%), catalytic activity (38.85%), cellular processes (38.22%), or single-organism processes (24.20%) (Figure 3D). For example, *Ghir_D13G021550* (*PLA2-BETA*) has been reported to be involved in pollen development, germination, and stomatal opening in response to light (Kim et al., 2011). Orthologs of *Ghir_A07G004250* (*AT4G32280.1*) have been reported to be involved in the regulation of indoleacetic acid (IAA) signaling (Shimizu et al., 2016) and have ovule-specific expression at 0 DPA and 1 DPA (Supplementary Figure S5). In addition, six genes in the Dt subgenome (*Ghir_D01G001790, Ghir_D13G021810, Ghir_D13G022780, Ghir_D13G023170, Ghir_D13G023060*, and *Ghir_D13G023090*) were shown to be involved in response to stimulus, which is consistent with previous reports (Liu et al., 2012; Su et al., 2020). In addition, some genes were involved in cellular component organization, organelle part, biological regulation, and cell part, with proportions ranging from 3.18 to 13.38% (Figure 3C). Specifically, *Ghir_D13G023010* (*RHIP1*) encodes a protein predicted to have a three-stranded helical structure, which has been previously shown to modulate early seedling development in *Arabidopsis* (Huang et al., 2015).

### Two Candidate Genes Pleiotropically Increase BW in Cotton Accessions

**Figure 4:**
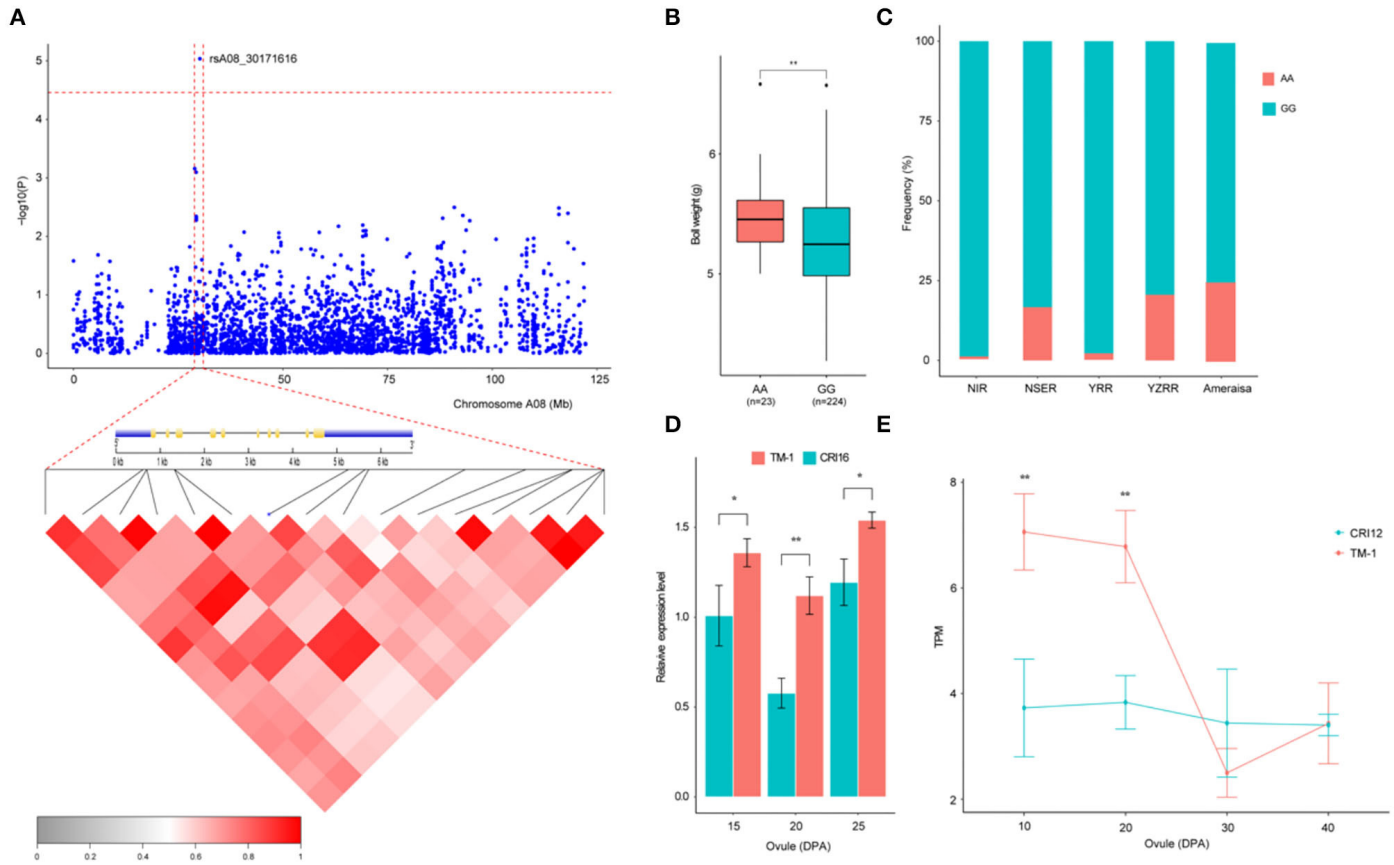
Variation analysis of the boll weight-related gene *Ghir_A08G009110* on candidate region. **(A)** Local Manhattan plots for BW-related genes on chromosome A08 and LD heatmap for the candidate region within the peak region of rsA08_30171616, including the exon–intron structure of *Ghir_A08G009110*. **(B)** Box plots for BW between the two haplotypes mentioned above (** *P* < 0.01, * *P* < 0.05). **(C)** Differentiation of the genetic diversity distribution of the favorable haplotype for rsA08_30171616 in five geographic areas. **(D)** Expression level analysis of *Ghir_A08G009110* between “TM-1” (red) and “CRI16” (green) during ovule developmental stages (15, 20, and 25 DPA) by qRT-PCR (** *P* < 0.01, * *P* < 0.05). **(E)** Expression abundance analysis of *Ghir_A08G009110* between “TM-1” (red) and “CRI12” (green) during ovule developmental stages (10, 20, 30, and 40 DPA) by RNA-seq (** *P* < 0.01, * *P* < 0.05).

Previous studies have indicated that QTLs for BW were widely distributed on all the chromosomes of cotton, but few QTLs mapped to chromosome A08 (Said et al., 2015; Li et al., 2016; Zhang et al., 2016). In this study, a novel QTL with a significant SNP (rsA08_30171616) on chromosome A08 exhibited the strongest association with BW, explaining 9.38% of the PV in two environments and the BW-BLUP (Figure 4A). This SNP has two haplotypes AA and GG, which led to the accessions carrying the GG haplotype having a significantly lower BW than those carrying the AA haplotype in nine environments (*P* < 0.05) (Figure 4B). In addition, to gain insight into the geographic distribution of the favorable haplotype (AA) for rsA08_30171616, the 290 cotton accessions were divided into five groups: NIR, NSER, YRR, YZRR, and Amerasian. NIR and YRR had a high proportion of the lines (Figure 1B) and showed an extraordinarily low AA frequency (Figure 4C), while the lines obtained from YZRR and Amerasian had a relatively high frequency of the favorable haplotype (>20%). We further performed an LD analysis of the significant SNP rsA08_30171616, and only one gene, *Ghir_A08G009110*, in the LD block was found in this region (Figure 4A). The quantitative reverse-transcription PCR (qRT-PCR) analysis and RNA-seq data showed that *Ghir_A08G009110* had higher expression levels in “TM-1” (BW = 6.18 ±0.83 g) carrying the AA allele than in “CRI12” (BW = 5.28 ±0.59 g) and “CRI16” (BW = 5.08 ±0.97 g) with GG allele during ovule development stage (Figures 4D,E). Through the above empirical results, we inferred that *Ghir_A08G009110* on chromosome A08 has potential role responsible for improving BW and may be beneficial to cotton breeding.

**Figure 5:**
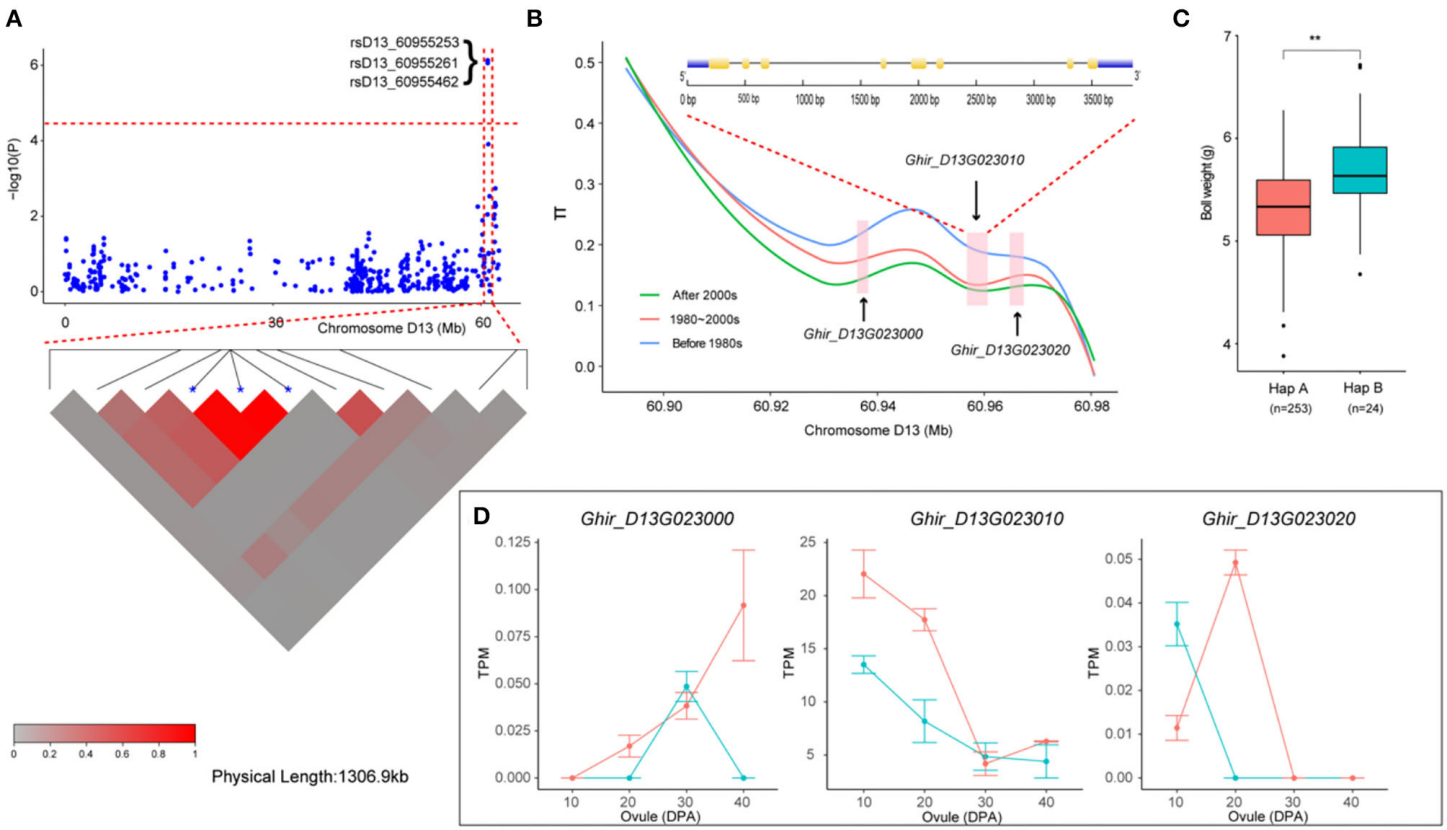
Variation analysis of the boll weight-related gene *Ghir_D13G023010* on candidate region. **(A)** Local Manhattan plots for BW-related genes on chromosome D13 and LD heatmap for the candidate region within the peak region of rsD13_60955253, rsD13_60955261, and rsD13_60955462. **(B)** Genetic diversity across the three populations and exon–intron structure of *Ghir_D13G023010*. **(C)** Box plots for BW of the two haplotypes mentioned above (** *P* < 0.01, * *P* < 0.05). **(D)** Expression abundance analysis of *Ghir_D13G023010* between “TM-1” (green) and “CRI12” (red) during ovule developmental stages (0, 10, 20, 30, and 40 DPA) by RNA-seq.

We then focused on a stable QTL on chromosome D13 (Figure 5A). Two SNPs and one InDel in this interval were stably associated with BW in six environments and with BW-BLUP, which could explain the relatively high PV from 10.32 to 10.95% (Table 2). Notably, three genes (*Ghir_D13G023000, Ghir_D13G023010*, and *Ghir_D13G023020*) were observed and tightly linked within the candidate region (Figure 5B). Furthermore, we found that the genetic diversity of this interval decreased with the breeding period; cotton cultivars released before the 1980s were dramatically more diverse than the cultivars bred in the 1980–2000s, and the cultivars bred after the 2000s showed the lowest diversity. These three elite alleles generated two haplotypes (HapA and HapB) in this LD block. Among them, rsD13_60955462 was located in the 3' UTR of *Ghir_D13G023010*. Varieties carrying HapB exhibited a higher average BW than those carrying HapA (Figure 5C). The RNA-seq data showed that *Ghir_D13G023010* had higher expression abundance level in the low-BW variety “CRI12” than in the high-BW variety “TM-1” compared with the other two genes during ovule development from 10 to 40 DPA (Figure 5D). The qRT-PCR analysis also showed that *Ghir_D13G023010* had higher expression levels in low-BW variety “CRI16” than in the high-BW variety “TM-1” during ovule development (Supplementary Figure S6). Thus, we inferred that *Ghir_D13G023010* is a novel gene that influences BW in cotton by negative regulation.

## Discussion

### Accurate Identification of SNPs and InDels

GWAS has become a commonly used method to identify elite allelic variation and candidate genes for important agronomic traits in cotton breeding and improvement (Fang et al., 2017; Wang et al., 2017; Ma et al., 2018). However, accurate genome sequence information enables the exploration and utilization of key genes that control important agronomic traits. It has been over 10 years since the first cotton genome sequence was published (Paterson et al., 2012; Wang et al., 2012). Since then, the number of cotton genomes sequenced has increased continually via multiple research studies due to the improvement in sequencing technologies in terms of cost, accuracy, and speed. The high rate at which genome sequences are becoming available is due to the development of next-generation sequencing (NGS), third-generation sequencing (TGS), and chromosome-scale scaffolding tools (Bio-Nano and Hi-C), with contig N50 values ranging from 0.11 Mb to 13.15 Mb in multiple upland cotton accessions (“TM-1,” “NDM8,” and “CRI24”) (Yu et al., 2014). A previous study demonstrated that the development of different reference-quality genomes could facilitate the investigation of novel variation and found new genes that were not discovered in previous SNP/InDel-based association analyses for important agronomic traits. For example, in maize, Tao et al. (2019) uncovered a novel causal mutation with an 8.9-kb insertion of a grain-size QTL (qHKW1) in an RIL population with the assistance of the newly assembled “SK” genome (Tao et al., 2019). In this study, to obtain accurate genetic markers, we employed a reference genome with a contig N50 greater than 100 kb for SNP and InDel calling. Although there was no significant difference in mapping rate, the genome version of HAU_v1 had more high-quality SNP and InDel markers. This genome provided a genetic basis for us to find a novel BW-associated locus. It is worth noting that 73.68% of associated BW loci could be detected via the comparison of multiple genomes. Five loci (rsGhir_A06_26390257, rsGhir_A06_26390265, rsGhir_A06_26390284, rsGhir_A06_26390468, and rsGhir_A06_26390491) on chromosome A06 are unique to HUA and are likely due to the diversity within the species and the quality of the reference genome. Therefore, the development of multiple reference genomes would enable the integration of these resources into high-quality pangenomes and will provide a better understanding of genetic diversity and a comprehensive guiding principle for the further exploration and utilization of this diversity for cotton improvement.

### Comparison of GWAS Results With Previously Reported Results

BW is an important determinant of yield and profitability in cotton and is controlled by multiple genes. Indeed, cotton breeding has constantly focused on the improvement of BW. Thus far, most QTLs for BW have been identified based on linkage analysis in the CottonQTLdb by using traditional molecular markers (Said et al., 2015). In addition, due to the limitation of traditional markers with lower levels of polymorphism and distribution density, it is difficult to attain sufficient resolution for fine map-based cloning and direct identification of candidate genes. GWAS has become a popular and powerful method to detect variants associated with major agricultural traits (Su et al., 2016, 2018; Fang et al., 2017; Wan et al., 2017; Ma et al., 2018; Zhang et al., 2019a). However, few studies have dissected the genetic basis of BW in cotton via GWAS in combination with high-throughput SNPs and diverse accessions across multiple environments in recent years, and even fewer candidate genes have been reported. In this study, 290 upland cotton accessions that were widely collected worldwide were used to conduct GWASs using high-throughput SNPs and diverse environments over multiple years. In total, 19 significant loci were identified among six different cotton chromosomes (Table 2), including 16 SNPs and three InDels. The identification of cotton varieties with stable yield and wide adaptation across a range of environments is one of the important objectives of modern cotton breeding programs in China. Although BW has relatively high heritability (69.65%), still lower than other agronomic traits in cotton, including oil content (96.6%) (Zhao et al., 2019), fiber length (81%) (Zhang et al., 2019a), flowering time (79%) (Li et al., 2021), and resulting, only a few stable QTLs were identified in 19 significant loci. This indicates that the remaining QTLs are affected by environment or genotype-by-environment. Meanwhile, phenotypic variation analysis found the BW of cotton grown in Huanggang is lower than that in Shihezi and Anyang. It is mainly caused by the high temperature in summer and the excessive rainfall in the later stage of cotton growth at the Yangtze River basin, leading to the correlation coefficient of E7 and E8 with other environments (E1–E6, E9) being low. Furthermore, although the SNPs obtained by SLAF-seq technology can well cover the whole genome of cotton, it must be admitted that there are indeed fewer stable QTLs than those obtained based on resequencing of GWAS. Therefore, we could employ resequencing for GWAS analysis in further to obtain more reliable QTLs for BW. To screen QTLs with high precision, high stability, and small confidence intervals for MAS and gene cloning, we further compared our results with published studies based on SNP and SSR markers (Said et al., 2015). Eleven reliable and significant markers located on chromosomes A07, D01, D07, and D13 were reported in previous studies. Three SNPs (rsGhir_A07_6937342, rsGhir_A07_6937395, and rsGhir_A07_9574709) on chromosome A07 overlapped with the region i49554Gh, which was named *qGhLP-c7* by Huang et al. (2017). rsGhir_D01_1229290, rsGhir_D01_1229442, rsGhir_D07_19492198, and rsGhir_D13_59526001 on chromosomes D01, D07, and D13 were mapped to regions adjacent to TM47842_TM47844, TM64105, and TM82005, respectively, as reported by Zhu et al. (2021). Most importantly, we also discovered a major QTL that was detected in multiple environments and with multiple BW-BLUP values and that could explain more than 10% of the observed PV. Furthermore, this region also overlapped with TM82122, as described by Liu et al. (2018), and narrowed the candidate region to 60.82–60.95 Mb on chromosome D13 containing three candidate genes. To date, few QTLs for BW on chromosome A08 have been identified in previous studies. Interestingly, a tightly linked region flanked by rsGhir_A08_30171616 on chromosome A08 was detected in two environments and with BW-BLUP values. This region contained only one gene (*Ghir_A08G009110*), which was not reported to control the boll weight of cotton in previous studies. Thus, these stable QTLs that are responsible for BW may have a significant effect on further yield improvement in cotton with appropriate BW.

### Candidate Genes Related to BW

It is known that BW is a complex quantitative trait controlled by many genes. Here, based on the association analysis, candidate gene expression analysis, and genetic diversity analysis of BW in 290 diverse cultivated upland cotton accessions, *Ghir_A08G009110* and *Ghir_D13G023010* on chromosomes A08 and D13, respectively, were identified as candidate genes for QTLs controlling BW in a natural population. Interestingly, *Ghir_A08G009110*, a unique candidate gene within the strong LD region 200 kb upstream and downstream of rsA08_30171616, encodes a protein containing ankyrin and DHHC-CRD domains in *A. thaliana* and is involved in root hair cell growth (Wan et al., 2017). We also discovered that the candidate gene *Ghir_A08G009110* in this region was highly expressed during the early stage of ovule development in the high-BW variety (Figures 4D,E). In addition, *Ghir_A08G009110* showed excellent potential for improving cotton yield and was not associated with other important agronomic traits in a previous QTL analysis (Said et al., 2015). Therefore, it is reasonable to postulate that *Ghir_A08G009110* is a new candidate gene for influencing BW in cotton. However, cotton accessions with rsA08_30171616-A had a much higher allele frequency than those with the potential superior alleles for *Ghir_A08G009110* in NESR and Amerasian, including accessions with a higher genomic proportion of some early core accessions. YRR and NIR, which contained mostly modern accessions, had a lower proportion of superior alleles for *Ghir_A08G009110* (rsA08_30171616-G). Thus, it is possible that the locus rsA08_30171616-A associated with excellent BW was screened out during the breeding process, so it is necessary to use rsA08_30171616-A as a tagging SNP in MAS of cotton lines to further improve yield.

Seed weight is also selected for during crop domestication, and understanding the genetic and molecular mechanisms controlling seed size has become an important research topic in plant science (Lin et al., 2014). Cotton is the largest economically important crop in the world, and breeders have expended a great deal of effort in improving the yield of cotton during long-term selection. Recently, *Ghir_D03G011310* was considered a candidate gene underlying the natural variation in cotton that controls early maturity in a natural population during long-term artificial selection, as stated in our previous report (Li et al., 2021). Furthermore, Wang et al. (2017) found many genes involved in the domestication of white fiber. However, the genes underlying the natural variation in cotton BW are still largely unknown. Here, we compared the genetic diversity of the region from 60.91 to 60.97 Mb on chromosome D13 containing *Ghir_D13G023010* in different breeding periods, and it was found that cultivars bred after the 2000s had lower genetic diversity than cultivars released before the 1980s and cultivars released in the 1980s−2000s. This result implied that with the continuous increase in cotton yield during the breeding process, this region is associated with artificial selection and with the increase in the BW of cotton. In addition, *Ghir_D13G023010* was the only *RHIP1* homolog in the cotton genome and was the best match with *Ghir_D13G023010* in the *Arabidopsis* genome. *RHIP1* is an uncharacterized conserved protein that participates in sugar signaling and plays significant role in negatively regulating seeding development (Huang et al., 2015). In particular, *Ghir_D13G023010* has highly expression abundance in the low-BW variety than in the high-BW variety (Figure 5D and Supplementary Figure S6). From the above results, we inferred that *Ghir_A08G009110* and *Ghir_D13G023010* were major candidate genes that may play an important role in influencing cotton boll weight.

## Data Availability Statement

The datasets presented in this study can be found in online repositories. The names of the repository/repositories and accession number(s) can be found in the article/Supplementary Material.

## Author Contributions

SY and ZF supervised the study and were involved in writing—reviewing and editing. LL was involved in funding acquisition, investigation, visualization, and writing—original draft. MT conceptualized the study and was involved in data curation and formal analysis. QL and CH designed software and data curation. DS validated the study. ZJ, GL, SZ, and GZ investigated the study. YZ designed software and visualized the study. All authors contributed to the article and approved the submitted version.

## Funding

This research was sponsored by the Program for Research and Development of Zhejiang A&F University (2021LFR005).

## Conflict of Interest

The authors declare that the research was conducted in the absence of any commercial or financial relationships that could be construed as a potential conflict of interest.

## Publisher's Note

All claims expressed in this article are solely those of the authors and do not necessarily represent those of their affiliated organizations, or those of the publisher, the editors and the reviewers. Any product that may be evaluated in this article, or claim that may be made by its manufacturer, is not guaranteed or endorsed by the publisher.
