# Supplementary material for: Detection of Stable Elite Haplotypes and Potential Candidate Genes of Boll Weight Across Multiple Environments via GWAS in Upland Cotton
Source: Front Plant Sci. 2022 Jun 13;13:929168. doi: 10.3389/fpls.2022.929168 (PMC9234699; doi:10.3389/fpls.2022.929168)
Supplement: Supplementary file 1 [file Data_Sheet_1.PDF]

Supplementary Figures

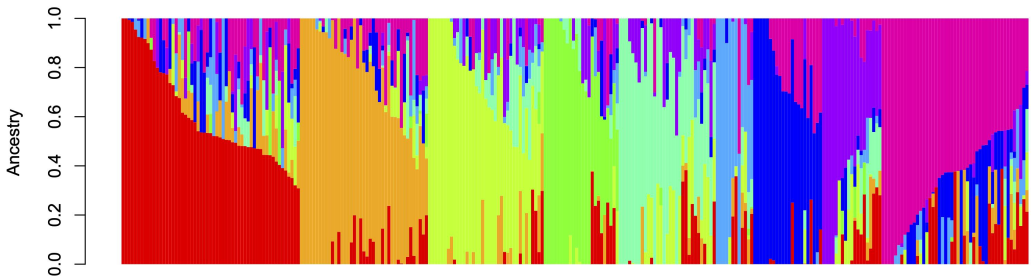

Figure S1: Structure analysis of GWAS population

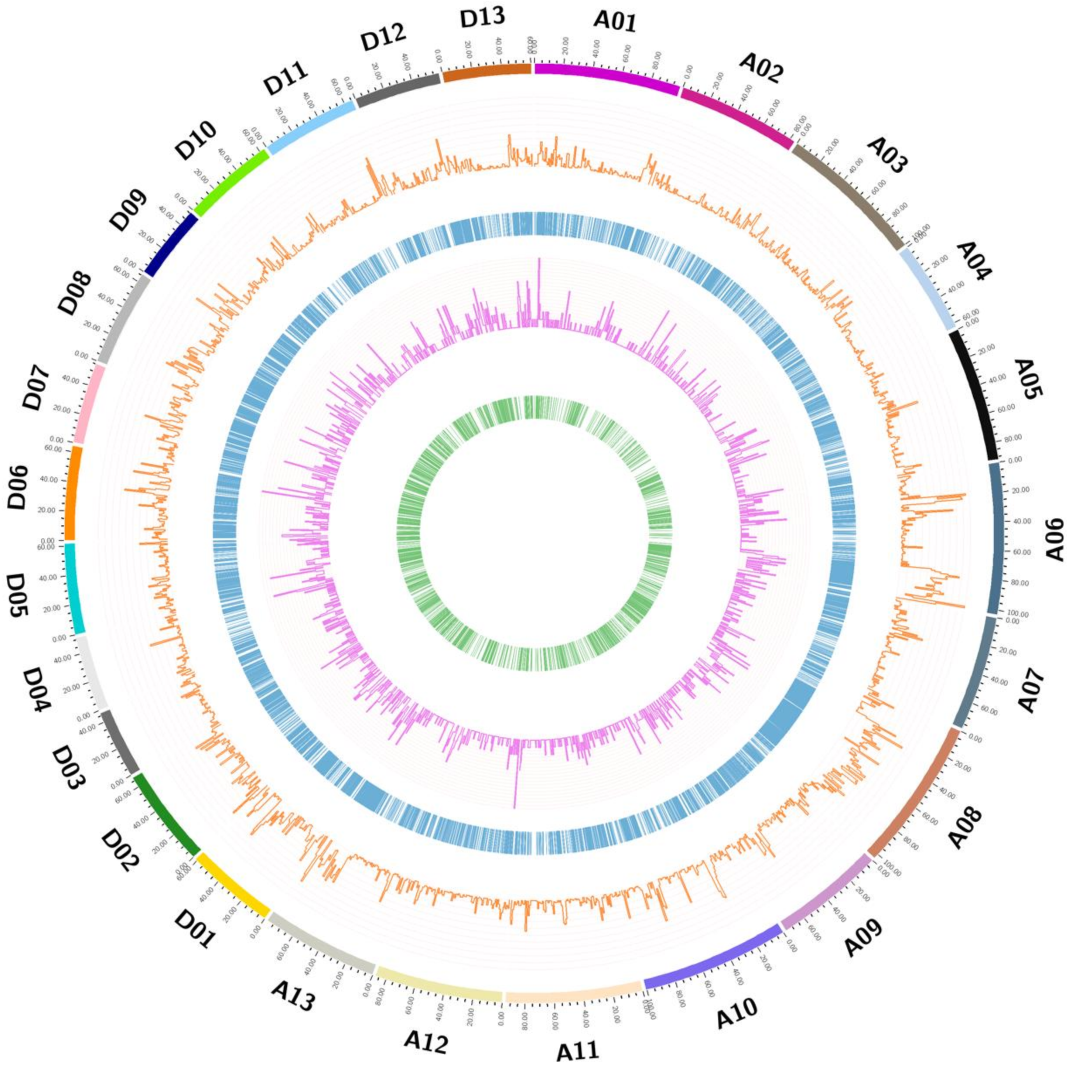

Figure S2: Circos plot showing SNP diversity across the 26 chromosomes of upland cotton; the chromosomes are numbered. The blue and green colors represent all the SNP and InDel marker distributions in different cotton genome regions, respectively. The orange and purple circles represent SNP and InDel densities, respectively

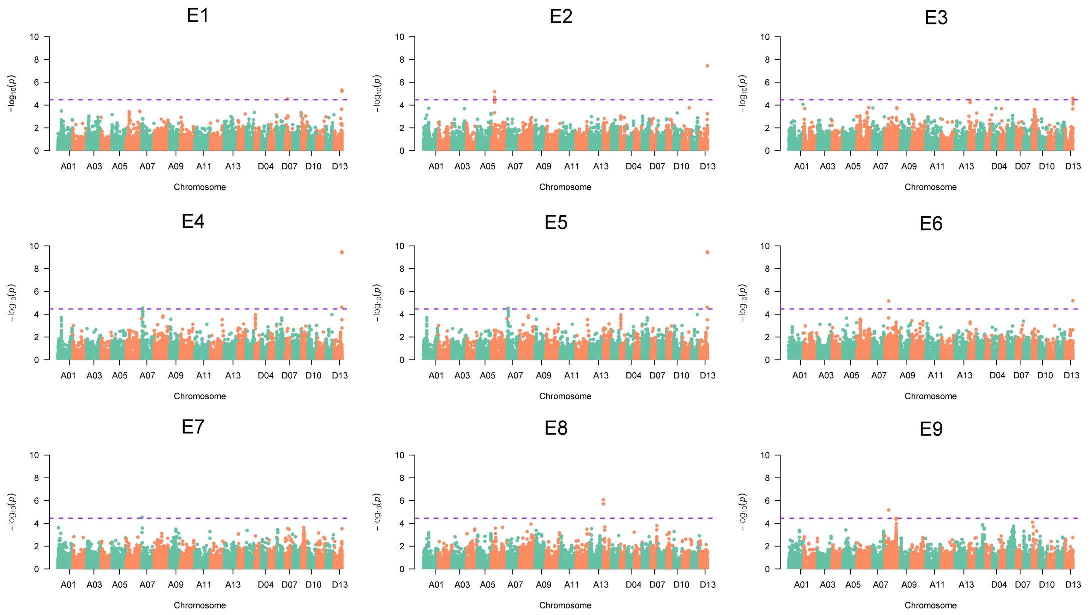

Figure S3: Manhattan plots for BW in each separate environment against SNPs; significant trait-associated SNPs are distinguished by purple lines

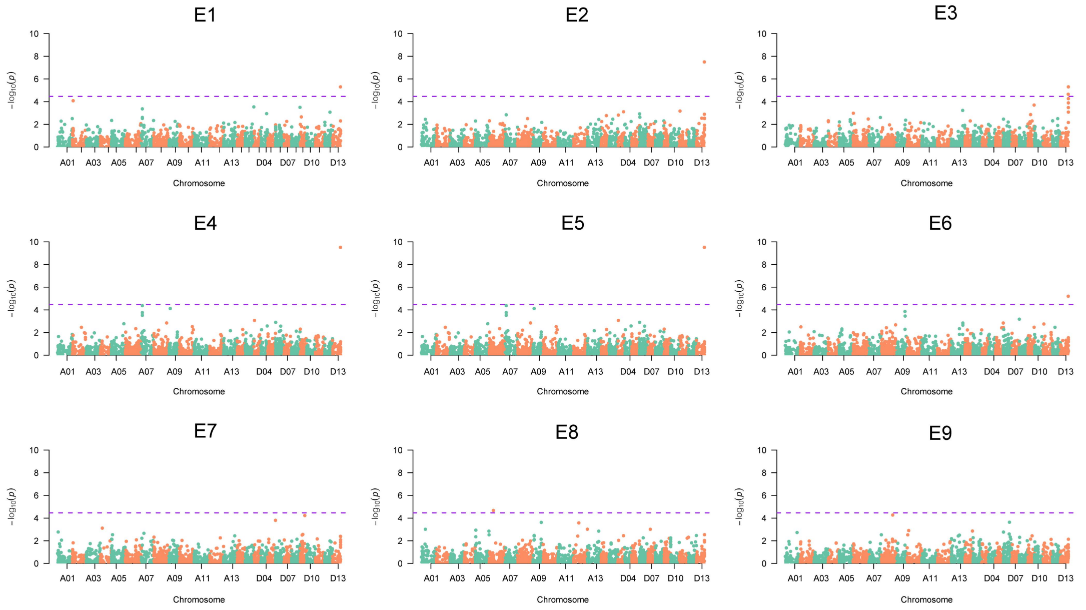

Figure S4: Manhattan plots for BW in each separate environment against InDels; significant trait-associated InDels are distinguished by purple lines

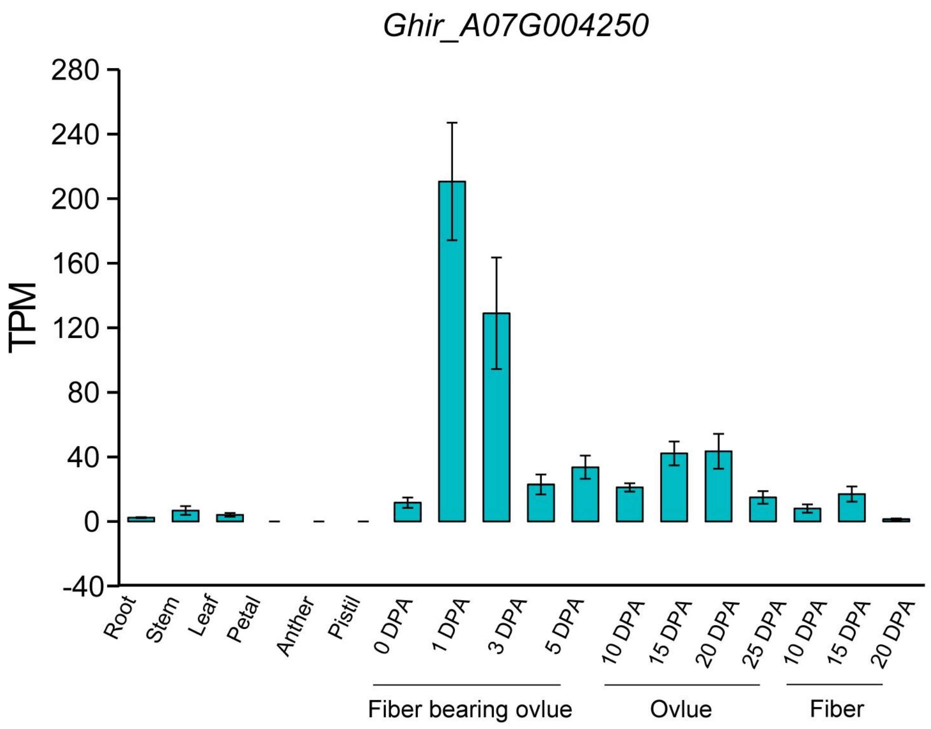

Figure S5: Expression level analysis of *Ghir_A07G0004250* in different tissues by RNA-seq

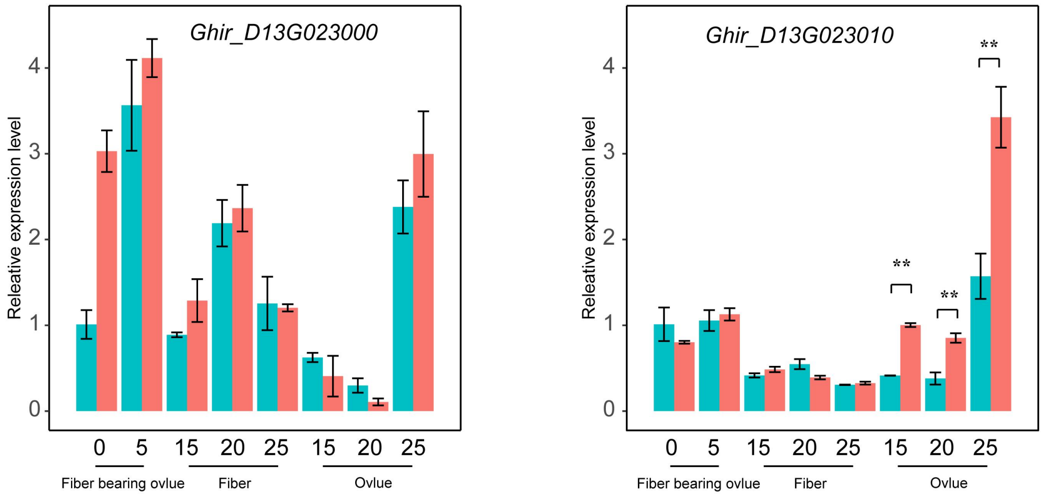

Figure S6: Expression level analysis of *Ghir_D13G023000* and *Ghir_D13G023010* in different tissues by qRT-PCR between 'CRI16'(red) and 'TM-1'(green)
